# Phlorotannin Supplement Improves Scopolamine-Induced Memory Dysfunction by Rescuing Synaptic Damage in Mice

**DOI:** 10.4014/jmb.2407.07009

**Published:** 2024-09-13

**Authors:** Minji Kim, Haeun Lee, Sangoh Kwon, Seungmok Cho, Min Young Um

**Affiliations:** 1 Division of Functional Food Research, Korea Food Research Institute, Wanju 55365, Republic of Korea; 2 Department of Food Biotechnology, University of Science and Technology, Daejeon 34113, Republic of Korea; 3 S&D Research and Development Institute, Cheongju 28156 Republic of Korea; 4 Department of Food Science and Technology/Institute of Food Science, Pukyong National University, Busan 48513, Republic of Korea

**Keywords:** Phlorotannin supplement, memory deficits, scopolamine, synaptic function, brain-derived neurotrophic factor

## Abstract

This study investigated the efficacy of a phlorotannin supplement (PS) in ameliorating scopolamine (SCO)-induced memory deficits in mice, focusing on synaptic function and the underlying molecular mechanisms. Male C57BL/6N mice were divided into six groups and treated with vehicle, donepezil (5 mg/kg body weight, (BW)), or PS (100, 250, or 500 mg/kg BW) for 6 weeks. Behavioral tests were conducted, followed by Golgi staining, immunofluorescence, and immunoblotting to assess synaptic protein expression and signaling pathways. Behavioral tests showed that PS administration significantly improved SCO-induced memory impairment and restored synaptic protein expression (synaptophysin, synapsin1, and postsynaptic density protein 95) in the hippocampus. Additionally, PS enhanced brain-derived neurotrophic factor (BDNF) signaling and activated the extracellular signal-regulated kinase/CAMP response element binding protein (ERK-CREB) pathway, essential for synaptic plasticity. Our findings demonstrate that PS mitigates SCO-induced memory dysfunction by protecting synaptic integrity and activating the BDNF-ERK-CREB signaling pathway, indicating the potential of PS as a natural intervention for treating memory deficits.

## Introduction

Aging and neurodegenerative diseases such as Alzheimer’s and Parkinson’s diseases are increasing globally owing to demographic transitions [1]. Most age-associated neurodegenerative disorders are characterized by progressive memory loss and learning deficits [2]. Epidemiological studies highlight a significant increase in the risk of dementia among individuals with mild cognitive impairment [3]. Cognitive decline, linked to aging and neurodegenerative pathologies, poses a significant barrier to a healthy life. Despite concerted efforts to discover pharmacotherapeutic agents that target cognitive decline, no drug can alleviate cognitive dysfunction. Therefore, exploring natural interventions that can attenuate or prevent cognitive degeneration is imperative.

Synaptic loss is closely associated with cognitive decline and is a hallmark of neurodegenerative diseases [4]. Reduced synaptic proteins, diminished synaptic density, and impaired synaptic plasticity contribute to cognitive decline [5]. Synaptophysin, found in all neuronal terminals, plays a crucial role in the exocytosis and endocytosis of synaptic vesicles [6]. Postsynaptic density protein 95 (PSD-95) supports memory and learning by facilitating calcium ion influx into neurons [7]. Furthermore, the accumulating evidence suggests that extracellular signal-regulated kinase (ERK) serves as a signaling molecule downstream of brain-derived neurotrophic factor (BDNF), and ERK phosphorylation activates cAMP response element binding protein (CREB) transcription factors, which regulate memory formation and synaptic remodeling [8]. Thus, maintaining homeostasis of synaptic plasticity is crucial.

Phlorotannins, polyphenolic compounds primarily found in brown algae, have garnered attention because of their potent antioxidant, anti-inflammatory, and neuroprotective effects [9-12]. Previous studies in mice have demonstrated that phlorotannins exhibit sedative-hypnotic effects through gamma-aminobutyric acid A-benzodiazepine receptors and improve sleep maintenance in individuals with sleep disturbances [13]. Phlorotannin supplements (PS) from *Ecklonia cava* have been approved by the Ministry of Food and Drug Safety in South Korea as functional ingredients for improving sleep quality. Although phlorotannins have been reported to be cognitive enhancers in vitro and in vivo, the underlying molecular mechanisms targeting synaptic functions have not yet been investigated.

Therefore, this study aimed to elucidate the effects of PS on scopolamine (SCO)-induced memory deficits in mice, with a focus on synaptic function. We hypothesized that PS would improve cognitive performance and mitigate synaptic damage caused by SCO. By investigating the molecular and cellular mechanisms underlying these effects, we provided an understanding of the therapeutic potential of PS in mitigating memory deficits and synaptic dysfunction.

## Materials and Methods

### Preparation of Phlorotannin Supplement

**Fig. 1:**
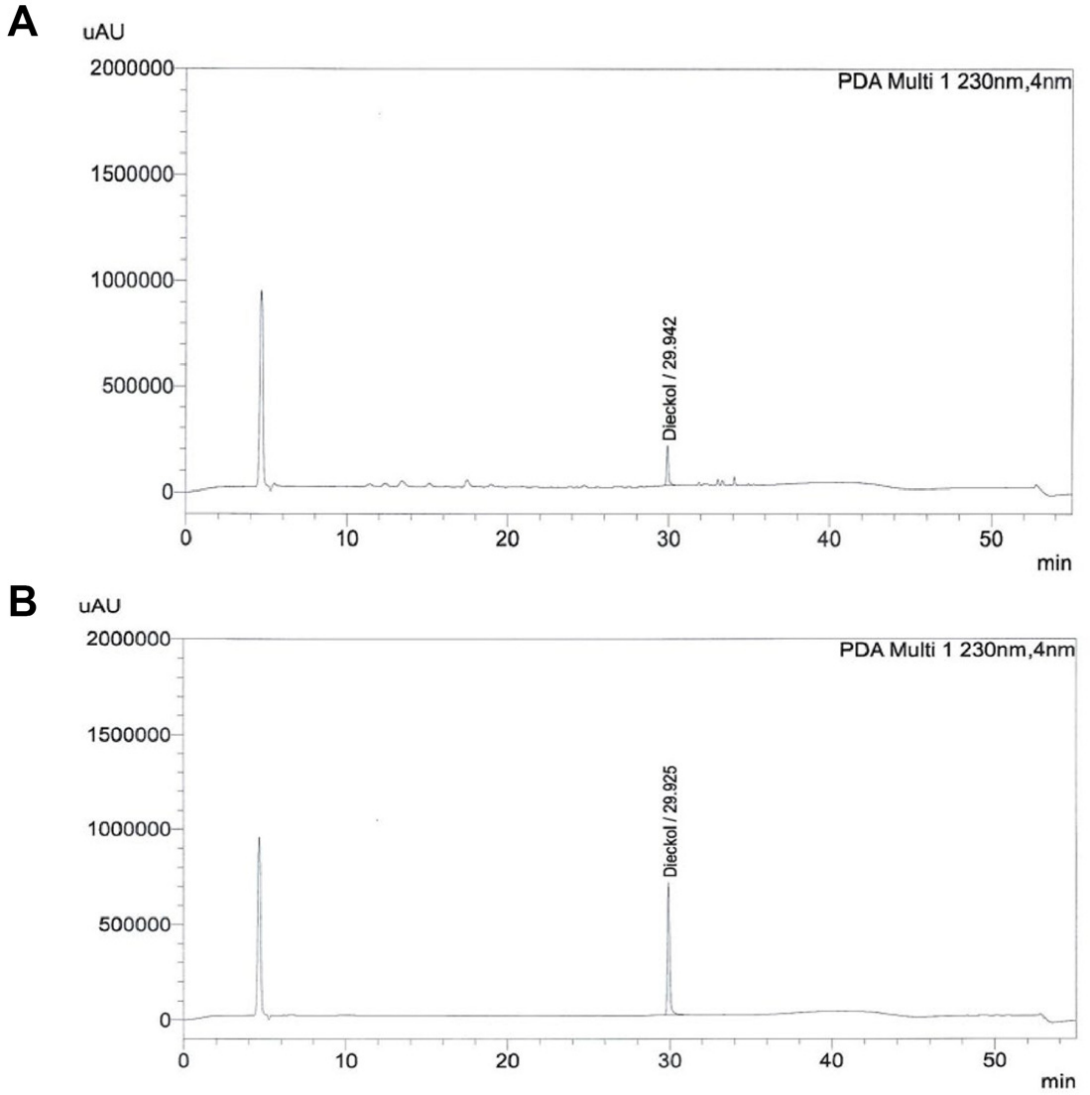
High-performance liquid chromatography chromatogram of (A) phlorotannin supplement and (B) dieckol as standard compound.

PS (Lot no. 240202-001) prepared from *E. cava* was provided by S&D Co. Ltd., (Republic of Korea). It was purified from *E. cava* using a synthetic adsorbent resin (Diaion HP-20; Mitsubishi Chemical Industries Ltd., Japan). *E. cava* was collected from the coast of Jeju Island, South Korea, between June 2023 and September 2023, and a voucher specimen was deposited at the herbarium of the Jeju Biodiversity Research Institute. The major components of PS include dieckol (67.5 mg/g), eckol (4.7 mg/g), eckstolonol (7.8 mg/g), triphlorethol A (9.9 mg/ g), fucodiphlorethol G (14.1 mg/g), and 6,6'-bieckol (5.2 mg/g) [13, 14]. PS was standardized with dieckol as the marker compound at a concentration of 60 mg/g extract (Fig. 1).

### High-Performance Liquid Chromatography

The concentration of dieckol was determined by a high-performance liquid chromatography system (HPLC; Agilent Technologies 1200 series, USA). The analytes were separated on a Supelco Discovery C18 column (4.6 × 250 mm, 5 μm; Supelco, USA). Each sample was analyzed at an ultraviolet wavelength of 230 nm. The HPLC system was operated at a flow rate of 0.7 ml/min in gradient mode, utilizing a mixed solution of (A) 0.01% acetic acid in 10% methanol and (B) 100% methanol. The gradient program over 55 min was as follows: 0–20 min, 15–30% B solution; 20–35 min, 30–85% B solution; 35–45 min, 85% B solution; and 45–55 min, 15% B solution. Peaks were identified by comparing the relative retention time, peak area percentage, and spectral data with those of the dieckol standard (SD-ECST-010, S&D Co., Ltd., Republic of Korea).

**Fig. 2:**
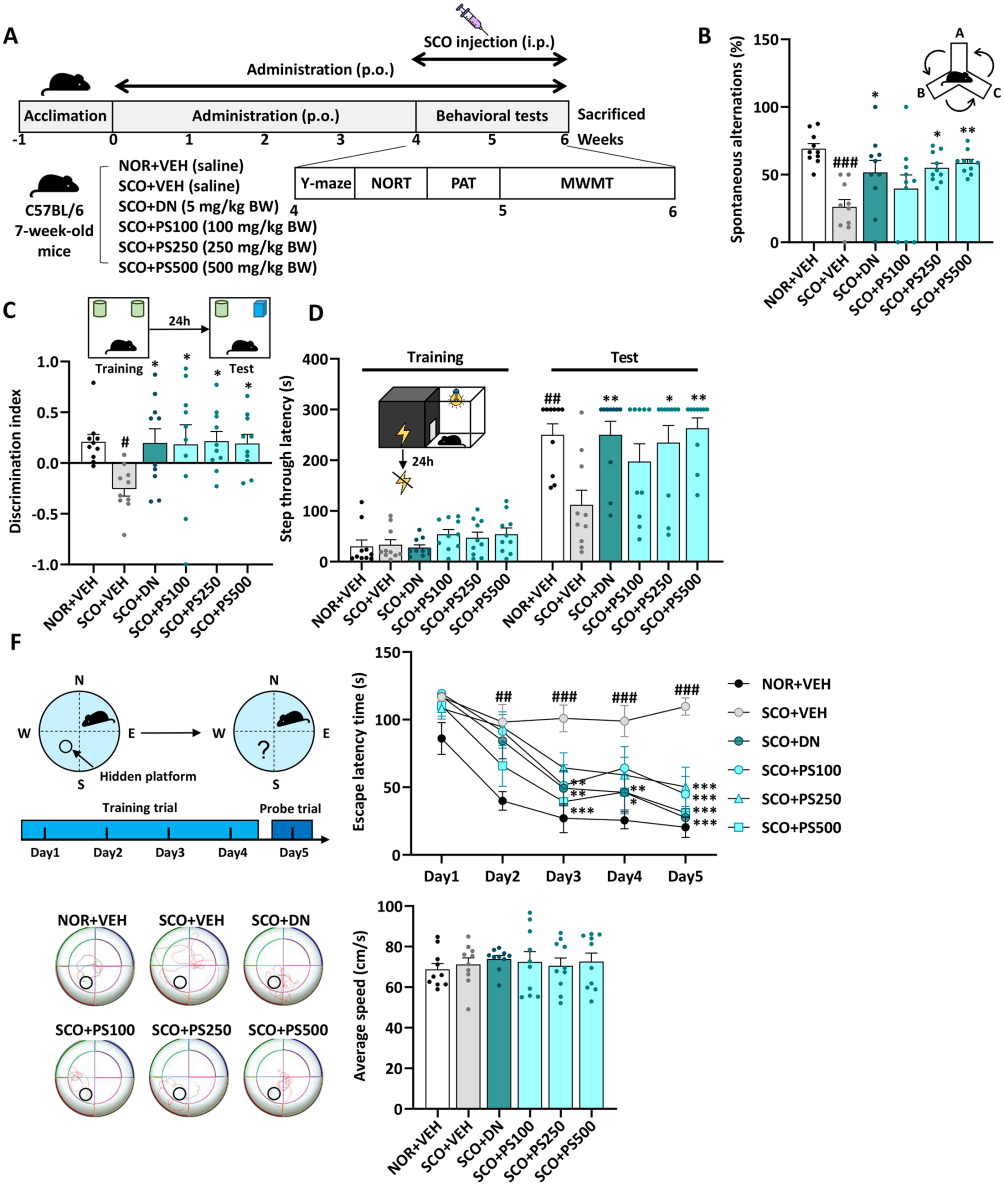
Phlorotannin supplements (PS) improves scopolamine (SCO)-induced memory deficits. (**A**) Experimental design and schedule. The mice were administered with or without PS (100, 250, or 500 mg/kg body weight (BW) per day, per oral) for 6 weeks, and subjected to behavioral tests. Before behavioral tests, the mice were injected SCO (1 mg/kg BW, intraperitoneally) (**B**) Spontaneous alterations (%) in the Y-maze test. (**C**) Discrimination index in the novel object recognition test (NORT). (**D**) Step through latency (s) in the passive avoidance test (PAT). (**E**) Experimental schedule of Morris water maze test (**MWMT**), escape latency time for 5 days. Representative images of tracking on the final test day (day 5) and average speed (cm/s) in the MWMT. # *P* < 0.05, ## *P* < 0.01, ### *P* < 0.001 vs. NOR+VEH group; * *P* < 0.05, ** *P* < 0.01, *** *P* < 0.001 vs. SCO+VEH group, one-way analysis of variance.

**Animals and experimental design.** All experiments were approved by the Institutional Animal Care and Use Committee of the Korea Food Research Institute (permission number: KFRI-M-23012). Male C57BL/6N mice, aged 7 weeks and weighing 18–22 g, were obtained from Koatech Co., Ltd., (Republic of Korea). After a one-week acclimatization period, mice divided into six groups (*n* = 10 per group): (1) NOR+VEH group, which was intraperitoneally injected with saline solution as vehicle (VEH) and orally administrated with VEH; (2) SCO+VEH group, injected with SCO and orally administrated with VEH; (3) SCO+DN group, injected with SCO and orally administrated with donepezil (DN) at a dose of 5 mg/kg body weight (BW); (4) SCO+PS100 group, injected with SCO and orally administrated with PS at a dose of 100 mg/kg BW; (5) SCO+PS250 group, injected with SCO and orally administrated with PS at a dose of 250 mg/kg BW; (6) SCO+PS500 group, injected with SCO and orally administrated with PS at a dose of 500 mg/kg BW. All doses aligned with those of previous studies [14-17]. All mice were intraperitoneally injected with SCO (1 mg/kg BW) 30 min before the behavioral test to induce memory impairment. After the behavioral tests were conducted, the mice were euthanized, and their brains and serum were collected for further analysis. The experimental schedule is shown in Fig. 2A.

### Behavioral Tests

**Y-Maze Test.** The Y-maze test was adapted from a previously described method to evaluate spatial and short-term memory in mice [18]. The apparatus comprises a black plastic structure with three arms, each forming a constant angle of 120°. Prior to testing, the plastic structures were cleaned with 70% ethanol and allowed to deodorize for a certain period. The experiment began with each mouse at the center point, where the three arms were crossed, and continued for 5 min. When the mouse head reached a point two-thirds the way through the arm, it entered the maze. The arms visited were recorded manually, and the total number of visits and alternations in which three different arms were visited consecutively were counted to measure the proportion of spontaneous alternations.

**Novel Object Recognition test (NORT).** The NORT was used to evaluate object recognition and memory [19]. The test was conducted in a transparent plastic box covered with opaque paper, to minimize the impact of external stimuli. Training and testing were performed over 2 days. On the first day, two identical objects were placed in a plastic box at specific locations, and the mice were allowed to explore the objects for 5 min. On the second training day, we replaced one object with another allowing the mouse to explore both objects freely. The exploration results were recorded using a camera, and the videos were analyzed using the SMART 3.0 video tracking system (Panlab, Spain).

**Passive Avoidance Test (PAT).** The PAT was performed as described by Um *et al*. [20]. The experimental apparatus (GEMINI, San Diego Instruments, USA) consisted of a dark chamber of the same size, a light chamber with a light bulb, and an automatic door connected to the two chambers. In the training trials, each mouse received an electric foot shock (0.5 mA) for 3 s when it entered the dark chamber from the lit chamber, after which the door was automatically closed. To assess memory, 24 h later, we measured the time between entering the light chamber and entering the dark chamber for 300 s under the same conditions, but no electric shock was applied to the dark chamber.

**Morris Water Maze Test (MWMT).** The MWMT was performed using a previously described method with minor modifications [21]. A circular tank containing opaque water (diameter, 100 cm; height, 135 cm) at 22 ± 1°C with powdered milk was divided into four sections: east (E), south (S), west (W), and north (N). The platform was placed 1 cm below the water surface between the western and southern sides. The test was conducted in two phases: training and probe trials. The training experiment lasted four days and involved training to find the platform within a specified time (120 s); if they did not find it, the mouse was left on the platform for 30 s to memorize the location. On the fifth day, the platform was removed, and the escape latency and swimming path length of each mouse were measured using the SMART polyvalent video tracking system (Panlab).

### Golgi Staining

Golgi staining was performed to visualize the hippocampal neuronal dendrites using a SuperGolgi kit (Bioenno Tech, LLC, USA). The perfused brain tissue was impregnated with the solution for 10 days at room temperature (RT) in the dark. After washing with phosphate-buffered saline (PBS) for 2 days, the impregnated brain tissue was sectioned at a thickness of 120 μm using a microtome (Leica Microsystems, Germany). The sections were washed again with PBS and stained overnight with a post-impregnation solution. The stained sections were briefly placed in the collection and mounting solution and air-dried on microscope coverslips. The samples were dehydrated in 70% ethanol four times for 5 min each, and the slides were cleared using xylene. After coverslipping, the slides were stored in a dark environment, and images were captured using a confocal microscope (Zeiss, Germany). Image analysis was performed using ImageJ software (National Institutes of Health, USA) to assess the morphology of the hippocampal neurons and the number of dendrites.

### Immunofluorescence Analysis

After the mice were anesthetized and perfused with 10% formalin. Subsequently, brain tissue was transferred to a tube containing formalin solution and dehydrated by immersion in 30% sucrose solution. The brain tissue was prepared by freezing it in ice cubes with optimal cutting temperature compounds. The frozen tissue was sectioned in the hippocampus region with a thickness of 20 μm using a microtome (Leica Microsystems, Germany). The sections were rinsed thrice for 5 min each with PBS containing Triton X-100 (PBST) and blocked for 1 h with PBST containing 10% goat serum. The sections were then incubated in a blocking solution containing primary antibodies against synapsin1 (A6442; Invitrogen), BDNF (ab108319; Abcam), and NeuN (MAB377; Millipore) at 4°C for 1 day. After rinsing three times with PBST for 5 min each, the sections were incubated with a solution containing secondary Alexa Fluor-conjugated antibodies (1:200) for 2 h in the dark at RT. The tissue was washed three times with PBST for 5 min each, after which the sections were mounted on slides and coverslipped using a 4',6-diamidino-2-phenylindole solution. The images were captured using a confocal microscope (Zeiss).

### Immunoblotting

The hippocampal tissue was homogenized in radioimmunoprecipitation assay buffer containing protease and phosphatase inhibitors. The lysate was then centrifuged at 13,000 g for 10 min at 4°C, and the supernatant was obtained. Protein concentrations were measured using the bicinchoninic acid protein assay kit. The quantified proteins were subjected to 10–12% sodium dodecyl sulfate-polyacrylamide gel electrophoresis and transferred to polyvinylidene difluoride membranes. Subsequently, the membranes were blocked with 5% skim milk for 1 h at 4°C, followed by overnight incubation at 4°C with primary antibodies against synaptophysin (4329S; CST), synapsin1 (A6442; Invitrogen), PSD-95 (2507S; CST), ERK (4695S; CST), p-ERK (4377S; CST), tropomyosin receptor kinase B ((TrkB) 4606S; CST)), p-TrkB (PA5-36695; Invitrogen), p-CREB (9198S; CST), CREB (9104S; CST), BDNF (ab108319; Abcam), and α-tubulin (3873S, CST). The membranes were then washed thrice for 10 min each with TBST and incubated with secondary antibodies for 1 h at RT. After another round of washing three times with TBST, the membranes were visualized using a chemiluminescence imaging system (LuminoGraph II EM, Japan). Protein intensity was quantified using ImageJ software (National Institutes of Health).

### Statistical Analysis

All data were represented as mean ± standard error of the mean, and differences were analyzed by one-way analysis of variance (ANOVA), followed by Tukey’s post hoc test using Prism 10 (GraphPad Software, Inc., USA). Statistical significance was set at *P* < 0.05.

## Results

### PS Improves SCO-Induced Memory Impairment

Mice were treated with VEH, DN (5 mg/kg BW), or PS (100, 250, or 500 mg/kg BW) for 6 weeks and subjected to various behavioral tests to determine the effect of PS on SCO-induced memory deficits (Fig. 2). In the Y-maze test, the SCO+VEH group reduced spontaneous alternations compared to the NOR+VEH group (*P* < 0.001). However, the administration of PS (250 or 500 mg/kg BW) significantly improved SCO-induced memory impairment (*P* < 0.05, *P* < 0.01, respectively) (Fig. 2B). As shown in Fig. 2C, the SCO+VEH group showed a lower discrimination index than the NOR+VEH group, indicating they had worse memory of objects. However, these phenomena were reversed by PS (250–500 mg/kg BW) administration, and in the SCO+DN group. In the PAT, the SCO+VEH group exhibited a more than 50% reduction in step-through latency compared to the NOR+VEH group. This reduced latency was significantly reversed in the SCO+DN group (*P* < 0.01). Similarly, PS administration (250–500 mg/kg) restored memory impairment induced by SCO treatment in a dose-dependent manner, demonstrating a potency comparable to that of the SCO+DN group (Fig. 2D). Fig. 2E shows representative swimming trajectories of these mice on the fifth day of MWMT. VEH-treated mice swam shorter distances to find the platform than SCO-treated mice. In contrast, PS administration performed better than SCO treatment alone. However, there was no difference in the average speed between the groups in the five-day platform exploration experiment for 5 days. These results indicated that PS administration may improve SCO-induced memory impairment.

### PS Ameliorates SCO-Induced Synaptic Defects

**Fig. 3:**
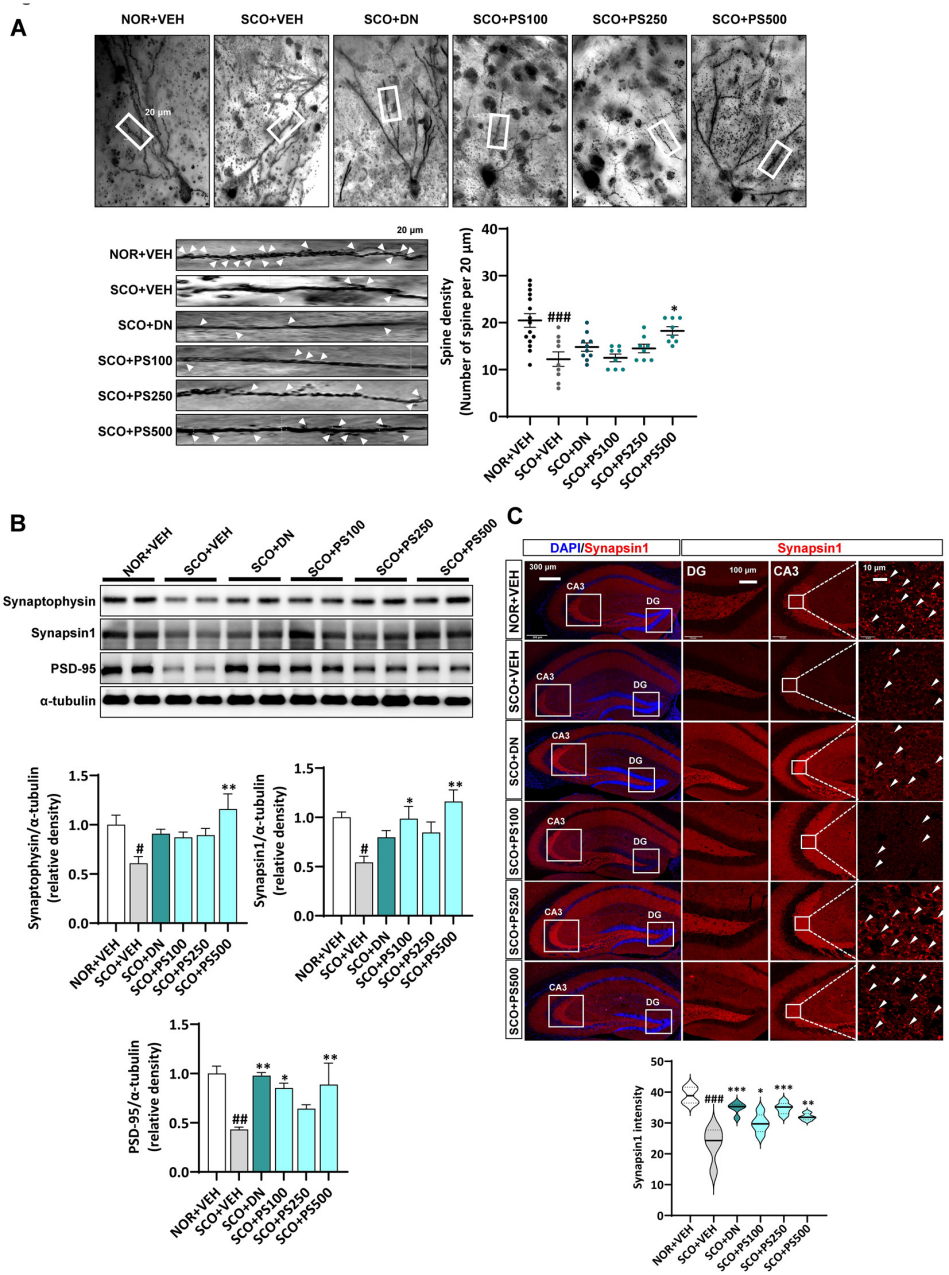
Phlorotannin supplements (PS) attenuates scopolamine (SCO)-induced synaptic abnormalities. (**A**) Representative dendritic images and dendritic spine number in hippocampal neurons of SCO-injected mice observed using Golgi-cox staining. (**B**) Immunoblotting analysis and quantification of synaptophysin, synapsin1, and postsynaptic density protein 95 (PSD-95) in the hippocampi of mice. (**C**) Representative confocal images of synapsin1 (red) and nuclei (blue) in the dentate gyrus (DG) and CA3 region of the hippocampus in SCO-injected mice. # *P* < 0.05, ## *P* < 0.01, ### *P* < 0.001 vs. NOR+VEH group; * *P* < 0.05, ** *P* < 0.01, *** *P* < 0.001 vs. SCO+VEH group, one-way analysis of variance.

We investigated how PS administration affects the dendritic spines and synaptic density of pyramidal cells in the hippocampus of memory-deficiency-induced mice using Golgi Staining. As shown in Fig. 3A, pyramidal cells in the CA1 layer of the hippocampus displayed significantly decreased dendritic spines in apical dendrites compared to those in the NOR+VEH group. Interestingly, Golgi staining also revealed a significant decrease in the average spine density in CA1 pyramidal cells in the SCO+VEH group but not in the NOR+VEH group. However, PS (500 mg/kg BW)-treated mice showed a remarkable recovery in spine density (*P* < 0.001), which was not observed in the SCO+DN group.

Next, we explored the expression of synaptophysin, synapsin 1, and PSD-95, which are markers of synaptic neurotransmission (Fig. 3B and 3C). Consistent with the findings of synaptic loss, the expression levels of these proteins were significantly reduced in the SCO+VEH group compared to those in the NOR+VEH group. In contrast, administration of PS restored the expression of these markers. Additionally, immunohistochemical analysis of synapsin1, a marker associated with synaptic function, showed that its intensity in the CA3 and dentate gyrus (DG) regions of the hippocampus was significantly lower in the SCO+VEH group than the NOR+VEH group (*P* < 0.001). However, this intensity increased in the mice administered with DN and PS (100, 250, or 500 mg/kg BW). These results demonstrate the significant role of PS administration in stabilizing synaptic protein expression and structure against SCO-induced synaptic defects.

### PS Activates the BDNF Signaling

**Fig. 4:**
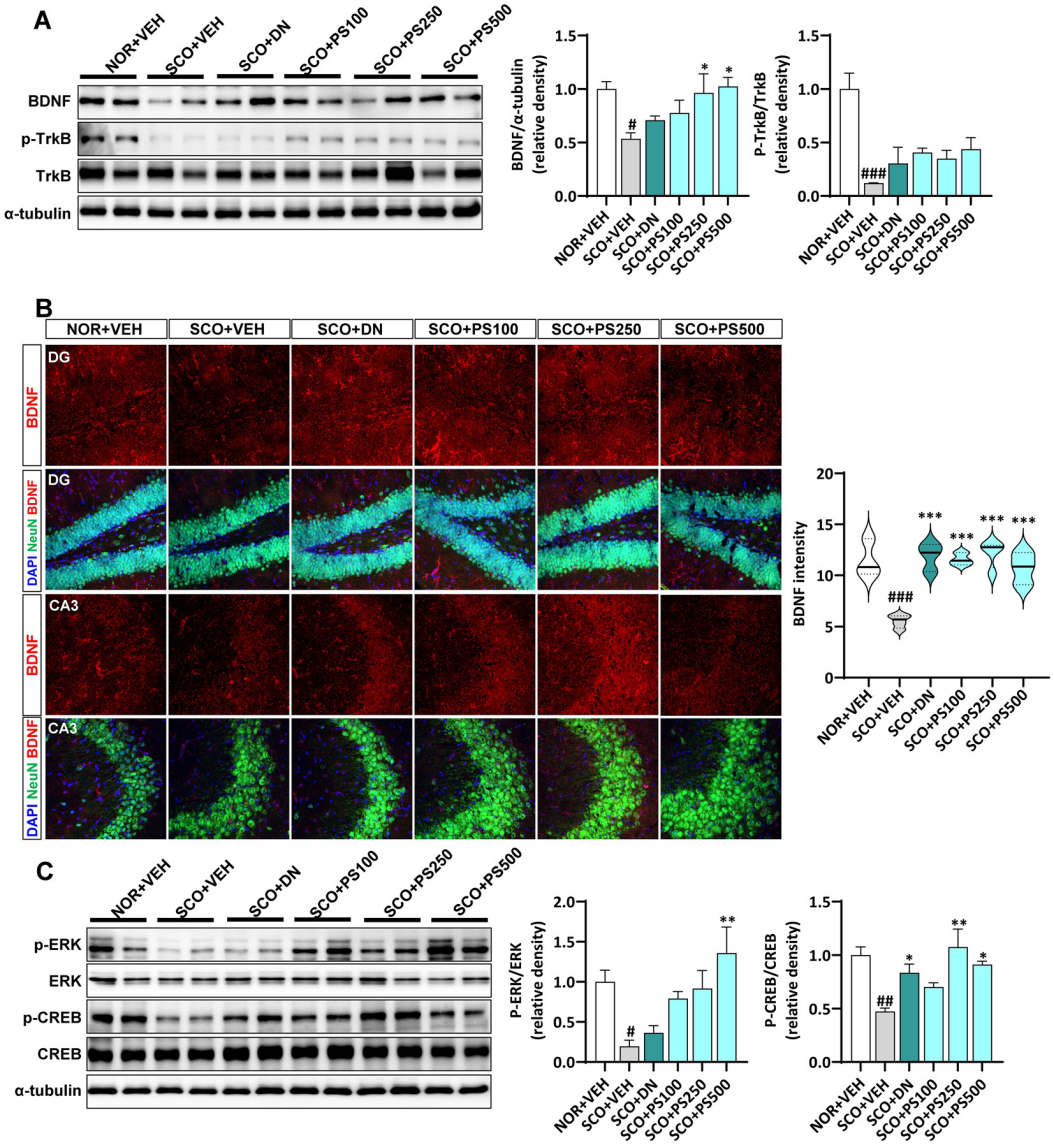
Phlorotannin supplements (PS) upregulates brain-derived neurotrophic factor (BDNF) and extracellular signal-regulated kinase (ERK)-cAMP response element binding protein (CREB) signaling pathway in the hippocampi of scopolamine (SCO)-induced mice. (**A**) Immunoblotting analysis and quantification of BDNF and the phosphorylation of tropomyosin receptor kinase B (TrkB) in the hippocampi of mice. (**B**) Representative confocal images of NeuN (green), BDNF (red), and nuclei (blue) in the dentate gyrus (DG) and CA3 region of the hippocampus in SCO-injected mice. (**C**) Immunoblotting analysis and quantification of the phosphorylation of ERK and CREB in the hippocampi of mice. # *P* < 0.05, ## *P* < 0.01, ### *P* < 0.001 vs. NOR+VEH group; * *P* < 0.05, ** *P* < 0.01, *** *P* < 0.001 vs. SCO+VEH group, one-way analysis of variance.

BDNF signaling via its transmembrane receptor TrkB plays a crucial role in the pathophysiology and treatment outcomes of cognitive deficit disorders [22]. Fig. 4A shows decreased BDNF and p-TrkB expression in the hippocampi of mice after SCO treatment. However, PS administration increased BDNF expression and TrkB phosphorylation in the hippocampi of SCO-treated mice. To corroborate these results, we confirmed the expression of BDNF in the hippocampal DG and CA3 regions using immunohistochemical analysis. The intensity of BDNF was significantly reduced in the SCO+VEH group compared to the NOR+VEH group, but this alteration was markedly restored by both DN and PS administration (Fig. 4B). Downstream ERK signaling plays a pivotal role in modulating BDNF transcription by stimulating CREB [23]. PS ameliorates SCO-triggered inactivation of ERK in the hippocampi of mouse brain. The expression of BDNF and the phosphorylation of its upstream regulator, CREB, were consistent with ERK expression. Overall, these findings suggest that the neuroprotective effects of PS are associated with the BDNF signaling pathway.

## Discussion

In the present study, PS ameliorated SCO-induced memory dysfunction by protecting against synaptic loss in the hippocampus via activation of the BDNF signaling pathway. SCO is a parasympatholytic agent that acts as a competitive muscarinic blocker [24]. SCO-induced memory loss serves as an experimental model of neurodegenerative diseases to assess the therapeutic potential of drugs [25]. Therefore, we used this animal model to evaluate the potential of PS and compare it to DN, a drug approved for treating dementia. SCO treatment significantly induced memory dysfunction compared to the NOR+VEH group in behavioral tests, and these deficits were recovered by PS administration. Numerous studies have explored the potential of PS to improve cognitive function in animal models of Aβ_1-42_-injected or PM_2.5_-induced cognitive decline [26, 27].

Phlorotannins, a group of polyphenolic compounds found in brown algae, have been extensively studied for their neuroprotective effects. Specifically, dieckol, one of the major components of phlorotannins, has been shown to enhance cognitive function by inhibiting acetylcholinesterase activity, thereby improving learning and memory in ethanol-treated mice [28]. Similarly, phloroglucinol, another monomeric component of phlorotannins, has been demonstrated to reduce memory deficits in 5×FAD mouse models [29]. Eckol and phlorofucofuroeckol, also present in PS, have been reported to possess neuroprotective properties that contribute to synaptic stability and cognitive enhancement [11]. Additionally, our previous study indicated that the phlorotannin-rich fraction from *Ishige foliacea* mitigated SCO-induced memory impairment via regulation of ERK-CREB-BDNF pathway despite difference in extraction methods and phlorotannin contents [20]. These findings suggests that cognitive improvements may not be may not be attributable to a single compound alone. Instead, they are likely due to the synergic effects of multiple phlorotannins working together to enhance cognitive function and protect against synaptic loss.

Synaptic plasticity, which encompasses the strength and adaptability of synapses, is critical for cognitive functions such as learning and memory [30]. In the present study, PS administration ameliorated synaptic abnormalities induced by SCO. Synaptophysin, synapsin1, and PSD-95 are essential synaptic health and neurotransmission markers. Among them, synaptophysin, found in neuronal terminals, plays a crucial role in the exocytosis and endocytosis of synaptic vesicles, while synapsin1 is involved in synaptic vesicle trafficking and neurotransmitter release [6, 31]. PSD-95 is a major scaffold protein that facilitates synaptic signaling and plasticity by anchoring key receptors and signaling molecules to synaptic sites. Our results showed that PS administration alleviated the SCO-induced reduction in protein expression, as confirmed by immunohistochemical analyses. Consistent with our findings, Kim *et al*. (2015) showed that phloroglucinol ameliorated the reduction in dendritic synaptic density induced by Aβ_1-42_ in primary hippocampal neurons and restored the reduced PSD-95 and synaptophysin protein in 5×FAD mouse model [29]. These findings underscore the importance of synaptic plasticity in memory function and highlight the role of PS in preserving synaptic integrity.

BDNF is also implicated in various neurological processes [32]. Mounting evidence suggests that BDNF signaling contributes to spin morphogenesis and is involved in synaptic protein synthesis and trafficking via the MEK/ERK pathway [33]. Additionally, activated CREB promotes BDNF expression, and BDNF promotes the activation of CREB through TrkB receptors [34]. BDNF, through its interaction with TrkB receptors, activates the ERK pathway, leading to the phosphorylation and activation of CREB [35]. Activated CREB promotes the transcription of genes involved in synaptic growth and plasticity, including BDNF, creating a positive feedback loop that enhances synaptic strength and resilience [36]. BDNF-mediated activation of the ERK pathway promotes the formation and maturation of dendritic spines, which are small protrusions on dendrites where synapses are located [37]. This process is essential for establishing functional neural circuits.

Furthermore, the ERK-CREB-BDNF pathway enhances synaptic transmission by increasing the synthesis and trafficking of synaptic proteins, thereby facilitating long-term potentiation, a long-lasting increase in synaptic strength following high-frequency stimulation of a synapse [38]. Thus, alterations in BDNF signaling could potentially lead to abnormal synaptic processes through protein translation factors, including CREB. Similar results were observed in our study, in which SCO induced inactivation of ERK and downregulation of CREB phosphorylation, both of which were efficiently reversed by PS administration. These findings suggest that PS alleviates SCO-induced memory dysfunction by protecting the dendritic architecture by activating the ERK-CREB-BDNF signaling pathway. However, this study has certain limitations. Although SCO animal models are widely used to study memory deficits, they may not fully replicate the complexity of human neurodegenerative diseases. Future studies should include other cognitive impairments and neurodegenerative models to validate our findings. Clinical trials are necessary to determine the potential of PS as a therapeutic agent for memory deficits.

In conclusion, our study highlights the therapeutic potential of PS in mitigating memory deficits by protecting against synaptic abnormalities and activating the ERK-CREB-BDNF signaling pathway, with the observed cognitive enhancements likely attributed to the synergistic effects of multiple phlorotannins, including dieckol, phloroglucinol, and eckol. These results, combined with the observed improvements in synaptic protein expression and dendritic spine density, provide a basis for the further exploration of PS as a natural intervention for cognitive decline.
